# Correction: Formation of plutonium(iv) silicate species in very alkaline reactive media

**DOI:** 10.1039/d2dt90053j

**Published:** 2022-04-06

**Authors:** Paul Estevenon, Thomas Dumas, Pier Lorenzo Solari, Eleonore Welcomme, Stephanie Szenknect, Adel Mesbah, Kristina O. Kvashnina, Philippe Moisy, Christophe Poinssot, Nicolas Dacheux

**Affiliations:** CEA, DES, ISEC, DMRC, Univ Montpellier Marcoule France thomas.dumas@cea.fr +33 4 66 33 92 05; ICSM, Univ Montpellier, CNRS, CEA, ENSCM Bagnols-sur-Cèze France nicolas.dacheux@umontpellier.fr; The Rossendorf Beamline at the ESRF CS40220 38043 Grenoble Cedex 9 France; Helmholtz Zentrum Dresden-Rossendorf (HZDR), Institute of Resource Ecology P.O. Box 510119 01314 Dresden Germany; Synchrotron Soleil, L'Orme des Merisiers, Saint-Aubin BP 48 F-91192 Gif-sur-Yvette Cedex France

## Abstract

Correction for ‘Formation of plutonium(iv) silicate species in very alkaline reactive media’ by Paul Estevenon *et al.*, *Dalton Trans.*, 2021, **50**, 12528–12536, DOI: 10.1039/D1DT02248B.

The authors regret an error in the data processing leading to a swap of information in Table 1 (on page 12529) between the above MWSA values and the below MWSA values. The corrected Table 1 is shown below.

**Table 1:** Conditions reported for the formation of actinide(iv) silicate colloids in carbonate ion rich reactive media and in carbonate ion free reactive media, where their formation was suspected (after ultrafiltration of the corresponding solutions)

Ref.	Actinide-silicate system	Conditions	pH	Colloid size	
				Below MWSA	Above MWSA
**Observation in carbonate ion rich reactive media**					
2	Th-Silicate	[NaHCO_3_] = 0.05 mol L^−1^	≥7	7–20 nm	
		[Th] = 10^−3^ mol L^−1^			
		Si/Th = 0.3–8			
3 and 23	U-Silicate	[NaHCO_3_] = 0.05 mol L^−1^	7–9.5	≤20 nm	
		[U] = 10^−3^ mol L^−1^			
		Si/U = 0.25–3			
24	U-Silicate	[NaHCO_3_] = 0.05 mol L^−1^	9–10.5	≤220 nm	1–10 nm
		[U] = 10^−3^ mol L^−1^			
		Si/U = 2–4			
4 and 25	Np-Silicate	[NaHCO_3_] = 0.1 mol L^−1^	7–9	≤250 nm	5 nm
		[Np] = 10^−3^ mol L^−1^			
		Si/Np = 0.7–8.6			
**Observation in carbonate ion free reactive media**					
20	Th-Silicate	ThO_2_·*x*H_2_O	6–12	Th solubility increase	
		[Si] = 10^−3^–0.15 mol L^−1^			
18	Th-Silicate	ThO_2_·*x*H_2_O	10–13.3	Th solubility increase	
		[Si] = 1.8 × 10^−2^ mol L^−1^			
15	Pu-Silicate	PuO_2_·*x*H_2_O	11–13.8	Pu solubility increase	
		[Si] = 10^−3^–10^−2^ mol L^−1^			

This mistake also led to a misinterpretation of the literature data reported in this table in the discussion. Indeed, the sentence beginning on line 25 of page 12529 (at the start of the penultimate paragraph of the Introduction section) is incorrect; the corrected text is:

“These colloids correspond to 1–20 nm particles at high silicate ion concentrations (above silicic acid mononuclear wall; [Si] = 2 × 10^−3^ mol L^−1^ (ref. 26)) and ≤200 nm agglomerates at lower concentrations”.

The sentence beginning on line 54 of page 12531 (in the third paragraph of the Results and discussion section) is also incorrect; the corrected text is:

“It is worth noting that no evidence of particle agglomeration was observed; that result is consistent with the results obtained for the other actinide silicate colloids above the silicic acid mononuclear wall.^4,24,25^”

These errors do not affect the overall conclusions of the paper.

The authors would like to apologize for these errors and any consequent inconvenience caused.

The Royal Society of Chemistry apologises for these errors and any consequent inconvenience to authors and readers.

## Supplementary Material

